# Clinical Characteristics and Outcomes of Patients with COVID-19 Infection and Acute Ischemic Stroke at Eka Kotebe General Hospital in Addis Ababa, Ethiopia

**DOI:** 10.4314/ejhs.v32i2.2

**Published:** 2022-03

**Authors:** Edom Gebremedhin Gebreegziabher, Nebiyu Getachew Mekonnen, Melaku Taye Amogne, Sebrina Ahmed Umer, Getahun Mengistu Takele, Dawit Kebede Huluka

**Affiliations:** 1 Department of Internal Medicine, College of Health Sciences, Addis Ababa University, Zambia St., Addis Ababa, Ethiopia; 2 Department of Neurology, College of Health Sciences, Addis Ababa University, Zambia St., Addis Ababa, Ethiopia

**Keywords:** COVID-19, acute ischemic stroke, cryptogenic stroke

## Abstract

**Background:**

Acute ischemic stroke has been reported to occur in a significantly higher number of COVID-19 patients as compared to healthy controls with variable proposed pathophysiologic mechanisms. To our knowledge, sufficient data regarding this subject is lacking in Ethiopia and the African continent at large. In this case series, we report the clinical characteristics and management of 5 cases with COVID-19 infection and acute ischemic stroke to shed light on the diagnostic and therapeutic challenges in resource-limited setups.

**Methods:**

This is a case series including data collected from the medical records of 5 participants with confirmed RT-PCR positive COVID-19 infection and radiologically confirmed acute ischemic stroke, admitted at Eka Kotebe General Hospital Intensive Care Unit (ICU) in Addis Ababa, Ethiopia from June 10, 2020, to November 04, 2020.

**Results:**

Cryptogenic stroke was documented in 4/5 participants included in this series with the most common vascular risk factors identified for stroke being hypertension and diabetes mellitus. The median time from onset of COVID-19 symptoms to the identification of stroke was 07 days. Two fifth of the participants in this series died during their ICU admission with the immediate cause of deaths reported to be related to the severe COVID-19 infection but not stroke.

**Conclusion:**

Cryptogenic stroke was documented in 4/5 patients in this series despite the presence of vascular risk factors for other stroke subtypes. The overall prevalence, subtypes, and outcomes of stroke in COVID-19 patients in Ethiopia and the African continent as a whole needs additional research to elucidate the local burden of the disease and define the predominant pathophysiologic mechanisms for stroke in COVID-19 in the region.

## Introduction

Coronavirus disease (COVID-19) is an infectious disease caused by a novel coronavirus designated as severe acute respiratory syndrome coronavirus-2 (SARS-CoV-2) (1). It was first reported as a cause of a cluster of pneumonia cases in Wuhan city in Hubei province of China at the end of December 2019 (1,2). WHO declared COVID-19 a global pandemic on March 11/2020 (1). As of August 15, 2021, more than 200 million confirmed cases and 4¶3 million deaths due to COVID-19 have been reported across 220 countries around the world (3).

COVID-19 has a multisystem involvement and manifestation with evolving pathophysiologic mechanisms. Among the neurologic manifestations of COVID-19 cerebrovascular diseases are relatively uncommon(5–9). Acute ischemic stroke in the setting of COVID-19 in hospitalized patients has been reported to occur in 0¶4 – 2¶7% (7–9, 11).

In this study, we describe the clinical characteristics and outcomes of patients with laboratory confirmed COVID-19 and radiologically confirmed acute ischemic stroke who were admitted at Eka Kotebe General Hospital Intensive Care Unit (ICU) from June 10, 2020, to November 04, 2020.

## Methods

**Study population**: Data were collected from the medical records of five participants with confirmed RT-PCR positive COVID-19 infection and radiologically confirmed acute ischemic stroke documented to occur during or near admission to Eka Kotebe General Hospital Intensive Care Unit (ICU), a dedicated COVID-19 treatment center in Addis Ababa, Ethiopia.

**Stroke assessment and classification**: All participants had ECG, echocardiography, and brain imaging with either CT or MRI. Additional laboratory tests included complete blood count, organ function and lipid panels, hemoglobin A1C and cardiac troponin. The stroke subtype for each participant was classified based on the Trial of ORG 10172 in Acute Stroke Treatment classification (10).

**Study variables**: The variables that were extracted from the participants' medical records include demographic data; clinical information such as known comorbidities and assessment of other stroke risk factors; and results of all laboratory tests and brain imaging. Additional data regarding the in-hospital treatment course, complications, and discharge outcomes were also collected from the charts.

**COVID-19 Diagnosis**: All of the participants included in the study were diagnosed to have COVID-19 by RT-PCR before admission to Eka Kotebe General Hospital.

**Ethical consideration**: Ethical approval (waiver) was obtained from the Research and Ethics Committee of Eka General Hospital (Ref No. E/1/150/5/38). All data are anonymized to identify participants.

## Results

Among the cases reported in this series, four are male and only one participant is female with a median age of 60 years. All of the participants are residents of Addis Ababa, Ethiopia. Traditional risk factors for ischemic stroke were documented in 3/5 participants, with hypertension being reported in all three participants, followed by diabetes mellitus and dilated cardiomyopathy in one participant each. Only one participant had a documented history of cigarette smoking. The demographic and clinical characteristics of the participants are reported in Table 1.

**Table 1:** Demographic and clinical characteristics of patients with COVID-19 and acute ischemic stroke admitted at Eka Kotebe General Hospital Intensive Care Unit (ICU) in Addis Ababa, Ethiopia from June 10, 2020 to November 04, 2020

	Demographic data	Clinical data	Clinical severity of COVID	Days from COVID-19 symptoms to stroke	Cardiac troponin (ng/ml)	Stroke subtype	Treatment	Outcome
Patient 1	53 / M	HTN, DCMP, prior cigarrete smoking (20 pack years)	Critical	07 days	< 0.1	Cardioembolic	Therapeutic UFH, Warfarin, Mannitol	Death
Patient 2	60 / M	-	Critical	14 days	-	Cryptogenic	Prophylactic UFH	Transferred to another hospital
Patient 3	60 / M	HTN	Critical	Not documented	< 0.03	Cryptogenic	Prophylactic UFH, ASA	Discharged
Patient 4	70 / F	HTN, DM	Severe	13 days	<0.01	Cryptogenic	Prophylactic UFH, ASA	Discharged
Patient 5	99 / M	-	Critical	Stroke symptoms precede the COVID by 15 days	0.02	Cryptogenic	Therapeutic LMWH, UFH, Mannitol	Death

ASA – Aspirin, DCMP – Dilated cardiomyopathy, DM – Diabetes Mellitus, HTN – Hypertension, LMWH – low molecular weight heparin, UFH – Unfractionated heparin

Productive cough and fatigue were the commonest COVID-19 symptoms seen in 4/5 participants each. Fever was documented in three participants included in this series. 4/5 participants had critical COVID-19 pneumonia and one had severe COVID-19 pneumonia requiring ICU admission with three of the participants requiring mechanical ventilation and the rest were managed with high flow oxygen support with face masks and noninvasive ventilation (NIV).

The median time lapse between the onset of COVID -19 symptoms and stroke onset is 1 week. Stroke was the reason for hospital admission in only one participant in this series with the rest of the strokes occurring during inpatient stay. The NIHSS scores at admission were not documented for all participants. Baseline cardiac troponin was documented normal in 4/5 participants. However, CRP and D-dimer were not determined in any of the participants. Ischemic stroke was confirmed by the use of non-contrast CT head and MRI in three and two of the participants respectively. 4/5 participants had multivessel territory involvement based on brain imaging patterns whereas one participant had single vessel territory ischemic stroke. Four of the participants in this series were found to have cryptogenic acute ischemic strokes based on the Trial of ORG 10172 in Acute Stroke Treatment classification, whereas one participant had presumed cardioembolic stroke.

All participants received IV broad spectrum antibiotics and ulcer prophylaxis. 4/5 participants were given dexamethasone whereas only one participant received chloroquine. Intravenous thrombolysis and mechanical thrombectomy were not done for any of the participants due to the unavailability of the services in the treatment center. Additional therapy with antihypertensive medications, diuretics, and insulin were given to the participants based on indication.

Acute kidney injury, hospital acquired pneumonia, and liver injury manifesting with transaminitis were reported in three participants each. Among the three participants requiring mechanical ventilation two participants underwent tracheostomy for prolonged intubation. Supraventricular tachycardias were the commonest cardiovascular complications documented in 4/5 participants in this series after admission to the COVID-19 treatment center.

Two fifth of the participants died during their ICU admission with the immediate cause of deaths reported to be related to the severe COVID-19 infection but not stroke, and three participants were discharged with improvement or transferred to another hospital. The median length of in-hospital stay was 24 days.

## Discussion

Variable pathophysiologic mechanisms are proposed to cause acute ischemic stroke in the setting of COVID-19 including hypercoagulability, vasculitis, cardiac dysfunction with low ejection fraction, and antiphospholipid antibody syndrome associated with the viral infection (14, 15, 17, 18).

In this case series, we report 5 participants with confirmed RT-PCR positive COVID-19 and imaging proven acute ischemic stroke. The occurrence of comorbidities and vascular risk factors for stroke such as hypertension, diabetes mellitus, and heart failure was found to be comparable to similar reports from Europe and the US, with hypertension documented in 3/5 patients in this series (8. 9, 11, 12).

In this case series, one participant was diagnosed to have a probable cardioembolic stroke due to the presence of dilated cardiomyopathy on echocardiography which is classified as a high risk source for cardioembolism (10). In a retrospective cohort study from New York, 7/32 patients with a concurrent diagnosis of COVID-19 and ischemic stroke were diagnosed to have cardioembolic stroke. The incidence of cardioembolic stroke in COVID-19 patients in the study was comparable to the contemporary and historical controls despite the higher prevalence of vascular risk factors in the latter groups than in COVID-19 patients (11).

In comparison in this case series, 4/5 participants were found to have cryptogenic stroke according to the Trial of ORG 10172 in Acute Stroke Treatment classification due to the presence of multiple risk factors for stroke in the background of an incomplete workup for all stroke etiologies. This rate is higher than reported in other parts of the world ranging between 35% – 65¶4% which might be attributable to the limited capacity of the medical center to carry out thorough workups for the stroke etiologies (7,8,11). Four participants in this series presented with COVID-19 symptoms before the recognition of stroke with a median time of 07 days which is comparable to similar reports in other countries of 07–24 days (8, 11–13, 16).

In line with the low mortality reported in Ethiopia due to COVID-19, the overall hospital mortality reported in these series of participants is lower than other similar reports despite the worse severity of illness in these groups of participants (8,9). However, since the residual neurologic function is not properly documented for the participants it is difficult to make further comparisons.

The small number of participants included in this series, lack of overall data regarding the prevalence, severity, and outcomes of patients with acute ischemic stroke and COVID-19 are difficult to make generalizations to the overall population. The resource limitation in the health system of the country with additional constraints imposed due to the pandemic is a significant barrier to the early identification, work up and treatment of patients with acute ischemic stroke and COVID-19 (19).

In conclusion, In this case series, cryptogenic stroke with multivessel territory involvement identified after hospitalization was documented at a possibly higher frequency as compared to reports from other parts of the world with a comparable prevalence of vascular risk factors, disease severity, and time to onset of neurologic deficit. However, the overall prevalence, subtypes, and outcomes of stroke in COVID-19 patients in Ethiopia and the African continent as a whole needs additional research to elucidate the local burden of the disease and define the predominant pathophysiologic mechanisms for stroke in COVID-19 in the region.
